# Rapid BMI Increases and Persistent Obesity in Small-for-Gestational-Age Infants

**DOI:** 10.3389/fped.2021.625853

**Published:** 2021-05-04

**Authors:** Dan Wu, Jianzheng Zhu, Xiulian Wang, Huiqing Shi, Yanyan Huo, Meiyan Liu, Fanfan Sun, Hongyan Lan, Chong Guo, Honghua Liu, Tingting Li, Lian Jiang, Xiangying Hu, Tianshu Li, Jing Xu, Guoying Yao, Guowei Zhu, Guangjun Yu, Jinjin Chen

**Affiliations:** ^1^Shanghai Children's Hospital, Shanghai Jiao Tong University, Shanghai, China; ^2^School of International and Public Affairs, Shanghai Jiao Tong University, Shanghai, China; ^3^Fujian Maternal and Child Health Hospital, Fujian, China; ^4^Jing'an District Maternal and Child Healthcare Center, Shanghai, China; ^5^Huang'pu District Maternal and Child Healthcare Center, Shanghai, China; ^6^Yang'pu District Maternal and Child Healthcare Center, Shanghai, China; ^7^Xu'hui District Maternal and Child Healthcare Center, Shanghai, China

**Keywords:** SGA, BMI changes, obesity, overweight, pediatric

## Abstract

**Purpose:** In order to compensate for the early intrauterine growth restriction, small-for-gestational age (SGA) infants have “catch-up growth” after birth. Increased caloric intake has been suggested for SGA infants conventionally. It is important to determine if the early growth rate of body mass index (BMI) is associated with risk of persistent obesity later in life. In this longitudinal cohort study, we assessed the BMI of a large cohort of children who were SGA at birth to determine their risk of persistent obesity at school age (6–7 years) due to excessive weight gain in the first 3 years of life.

**Methods:** We collected the height and weight data of 23,871 SGA babies. A polynomial function was used to fit the BMI-for-age z-score (BAZ) values of 0–6 years old SGA children and interpolate their growth trajectory. In addition, we screened out 6,959 children from 23,871 children to further evaluate the dynamic changes of early childhood BMI. We divided the school-age children into groups as non-obese (BAZ < 2) and obese (BAZ > 2), and determined the association between changes in BMI and school-age obesity.

**Results:** From the perspective of BMI distribution, the interpolated growth trajectory indicated that SGA children reaching overweight status or developing obesity by 3 years of age, continued to have obesity until school age (R^2^, 0.65; R^2^, 0.21). The retrospective analysis showed that children who were overweight and had obesity during school age had a high BMI from early age. By analyzing the changes in early BMI, we found that the fastest growth of SGA children occurred in the early infancy before 6 months and they continued to grow rapidly for a period of time. Interestingly, former SGA children who maintained a near overweight (1 < BAZ < 2) status before the age of 2 maintained an appropriate growth rate and usually did not develop obesity.

**Conclusions:** A rapid increase in BMI during early infancy in former SGA newborns leads to a persistent risk of obesity. The energy intake of SGA infants should appropriately meet the infants' growth needs and early BMI changes should be closely monitored for an optimal integrated management.

## Introduction

Nutritional diseases are common in children, however, over the years the disease spectrum has changed. On one hand, the prevalence of overweight and obesity in childhood remains high (1, 2). On the other hand, with advances in perinatal medicine, the survival rate of small-for-gestational-age (SGA) newborns has increased significantly. Studies have reported that the incidence of SGA in the United States was 15 per thousand term births in 2011, an incidence of around 29.9% from 2002 (3, 4). The incidence of SGA in Shanghai has increased from 2.74% in 2005 to 3.41% in 2010 (5). Surviving SGA newborns present risks for different conditions (6–8), especially for atypical growth patterns (i.e., catch-up growth). According to the hypothesis of fetal origins of adult disease, the risk of developing a nutritional disease caused by this atypical growth pattern is high. A child's risk of obesity continues to be present during adulthood i.e., most children with obesity continue to have obesity in adulthood (9, 10).

However, most clinicians recommend parents of SGA infants to focus on weight gain and generally advocate that they increase the energy intake of low-weight infants without moderation (11, 12). The long-term health consequences of excessive energy intake and the risk of obesity associated with rapid early growth are ignored. Therefore, it is important to determine whether SGA infants develop obesity early in life and whether a critical period of increased risk of obesity exists in such infants. An analysis of the infants' risk of developing obesity is important to facilitate the early integrated management of SGA newborns (13, 14). Studies predicting obesity in children based on their body mass index (BMI) have suggested the existence of a positive correlation between the two variables (9), but the research on low birth-weight (BW) infants is scarce. The pattern of infancy weight gain that leads to persistent obesity in former SGA newborns is unclear. In this study, we assessed the BMI of a large cohort of children who were SGA at birth to determine their risk of persistent obesity at school going age (6–7 years) due to excessive weight gain in the first 3 years of life.

## Patients and Methods

The Ethics Committee of Shanghai Children's Hospital Affiliated to Shanghai Jiaotong University School of Medicine approved this study (No. 2016R029-F01). The following were the inclusion/exclusion criteria were used for selection of participants:

### Inclusion Criteria

1. Full-term SGA infants born in Shanghai between April 2010 and October 2017 who underwent a physical examination at a maternity and child health center in a nearby district.
2. The parents of participant children providing relevant information and signing the informed consent form to have their children included in the cohort study at the 42-day medical examination.
3. In addition, the included children were to have at least one physical examination of height and weight when they were 0–3 years old and another examination when they were 6 years old (school-going age).

### Exclusion Criteria

Children with diseases that may adversely affect their growth and development, including premature birth, heart disease (more severe than grade II murmur), and asthma; endocrine diseases; nervous system diseases; moderate or severe rickets or other abnormalities affecting physical development; limb disability; acute diseases (such as pneumonia, dysentery, etc.,) that were resolved within the last month; fever for more than 7 days in the 2 weeks prior to enrolment or diarrhea occurring more than 5 times a day and lasting for more than 5 days.

**Figure 1:**
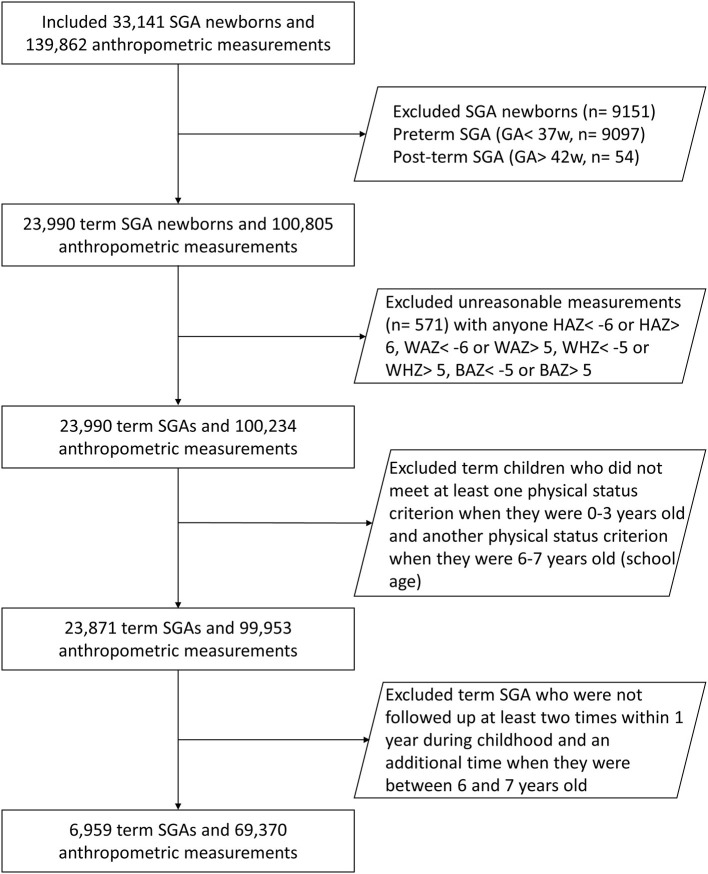
Study flow chart.

We categorized the children according to their BW percentiles (15, 16). We defined full-term SGA as an infant with a BW below the 10th percentile (P10) for infants of the same birth age and sex, with a birth age of 37 or more weeks (16). Figure 1 demonstrates the selection process of the study. In all, we included 23,871 full-term SGA infants in the study cohort.

### Physical Examination

Height: For measurements prior to 24 months of age, we used a standard measuring bed with the child in a supine position. The assistant positioned the child's head on the headboard with the child laying down. The person taking the measurement was on the right side of the child; an assistant used his left hand to hold the child's knee straight and his right hand to hold the foot plate in contact with the child's heel. The child's height was measured in centimeters, to the nearest tenth of a centimeter. Height was measured in standing position for 6 year old children.

Weight: The outerwear, shoes, hats, and diapers of the children were removed so that they were wearing only thin clothes (under a comfortable indoor temperature). The children's weights were recorded in kilograms (kg) using a standard measuring bed, with an accuracy of 0.01 kg.

### Quality Control

Before the formal investigation, we conducted a pilot investigation to overcome or minimize potential research problems in a timely manner and improve the accuracy of our study. All the data in our study is derived from formal investigations. All measurement operators were professional pediatric health care personnel with professional training and demonstrated proficiency in the unified technical standards and operating methods before the survey. All examinations were performed at a single medical facility, where the measuring instrument was inspected and calibrated every day before beginning the measurements. We replaced any contents in the survey (such as the individual's name) involved in privacy issues by a number in the subsequent data analysis. Members of the research group collected the research data. The data were reviewed for appropriateness, and any missing or incorrect items in the data were dealt with in a timely manner to improve the accuracy of the research data. We deleted data that could not be repaired to improve the credibility of the survey results.

### Statistical Analysis

The study was conducted in two parts with two different sample sizes and used two different ages to define of obesity.

For the first part of the analysis, we categorized the 23,871 full-term SGA infants according to age groups: 0 (0.0 to 0.9) years, 1 (1.0 to 1.9) years, 2 (2.0 to 2.9) years, and so on, until 6 (6.0 to 6.9) years in order to track BMI data. In cases with multiple follow-up data for a child in a certain age group, we selected the data from the single follow-up that corresponded to the age that was closest to the median age for the specific age group (e.g., 0.5, 1.5, or 2.5 years). We assigned SGA infants at 0–12 months to the following groups by BAZ (BMI-for-age Z-scores): underweight (BAZ < −2), normal weight (−1 to 1), nearly overweight (1 to 2) and overweight/nearly obese (BAZ > 2) (9). This grouping is not based on the diagnostic criteria, but the data is divided into 4 groups according to different BAZ values. Since there are almost no children who can be diagnosed with obesity according to the BAZ criteria for children aged 0–12 months, we classified children with overweight and obesity into a singular “overweight/nearly obese” group so as to make the BAZ development trajectory of SGA children more accurate. We then used a polynomial function to fit the BAZ values of these four groups of SGA children aged 0–6 years. After the data fitting, we interpolated the fitted curve and chose the cubic Hermite interpolation polynomial, which made the fitted curve smoother thereby stimulating their growth trajectory. We also compared the annual trends in the variation of BMI between these groups and explored a possible association between early rapid weight gain and persistent obesity in the four groups *via* plotted graphs (9).

In the second part of the analysis, we extracted data of children followed up at least two times within 1 year during childhood and an additional time when they were between 6 and 7 years old in order to assess the effect of annual changes in the BAZ (Figure 1). Data of 6,959 children fulfilled this criteria from the entire cohort of 23,871 SGA children. These children were grouped according to the developmental outcome of SGA during the school-age period, and the trend of BAZ change and the distribution of BAZ change rate were retrospectively observed. The grouping basis here was according to the WHO guidelines as non-obese (BAZ < 2) and obese (BAZ > 2).

## Results

### Basic Information on the Participants

**Figure 2:**
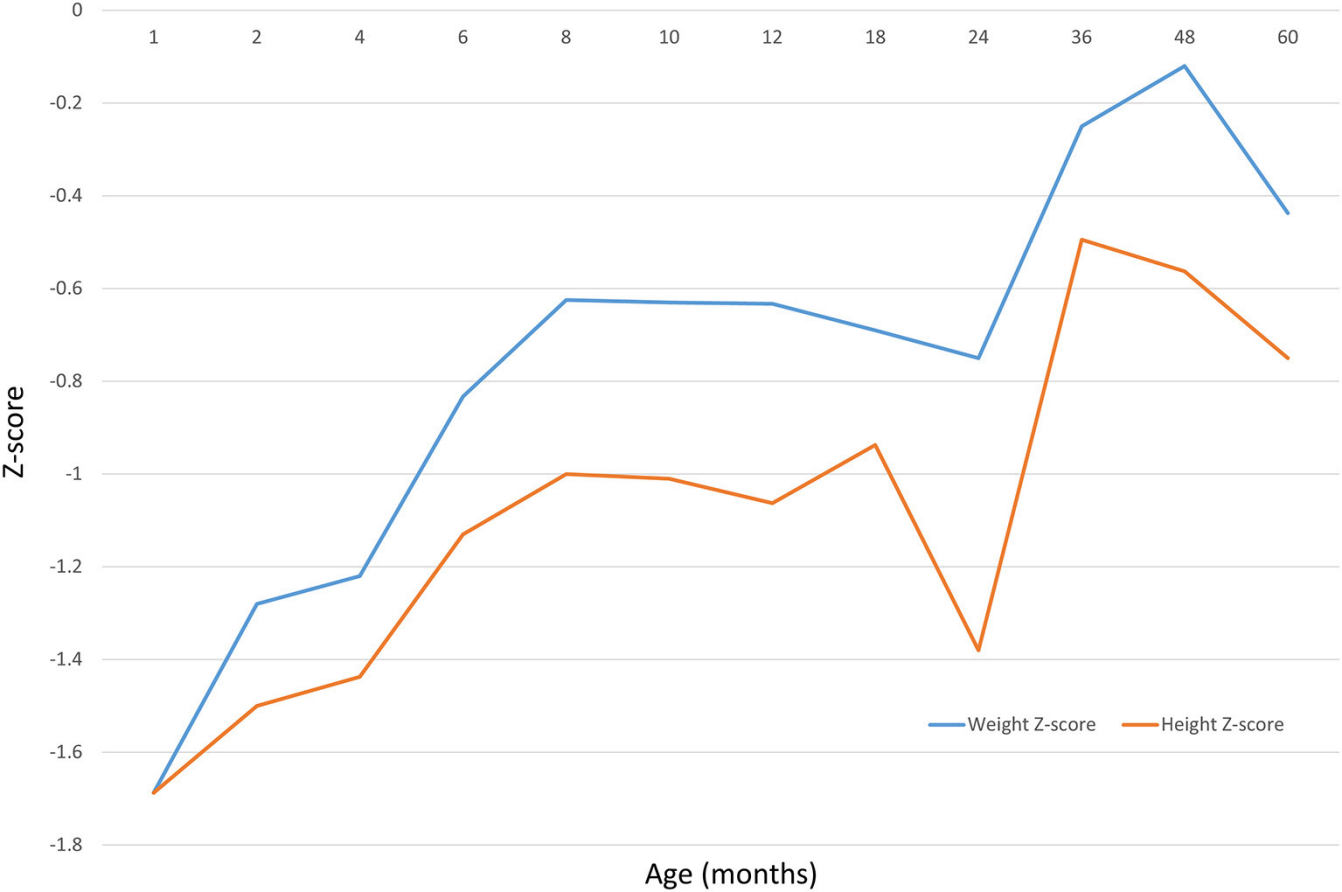
Data of 23,871 children shows the height (body length) and weight z-scores at different age.

Baseline details of all study participants is presented in Table 1. The changes in height (body length) and weight z-scores at different ages of the included children is presented in Figure 2. The results show increasing trends for both the SGA newborns' weight and height (length) z-scores. However, the scores always remained below the standard median. The increasing trend for height was not as high as that for the body weight, suggesting that SGA infants may have an obesity tendency due to excessive weight gains.

**Table 1:** Differences between the SGA babies included and excluded.

	**Included SGA babies in second analysis**	**Excluded SGA in second analysis**	***P***	**Total**
Number [*n* (%)]	6,959 (29.15)	16,912 (70.85)	–	23,871 (100)
**Sex [*****n*** **(%)]**				
Male	2,529 (36.34)	5,731 (33.89)	0.06	8,260 (34.6)
Female	44,30 (63.66)	11,181 (66.11)		15,611 (65.4)
Gestational age (GA, weeks)	39.06 ±1.13	39.13 ± 1.09	0.02	39.02 ± 1.11
Birth weight (BW, kg)	2.57 ± 0.16	2.58 ± 0.17	0.07	2.54 ± 0.18
**Residence [*****n*** **(%)]**			0.29	
Urban	1,404 (20.18)	2,846 (16.83)	–	4,250 (17.8)
Rural	5555 (79.82)	14,066 (83.17)	–	19,621 (82.2)

*GA and BW are shown in mean ± SD*.

### Growth Trajectory From Early Infancy to School-Age Children

**Figure 3:**
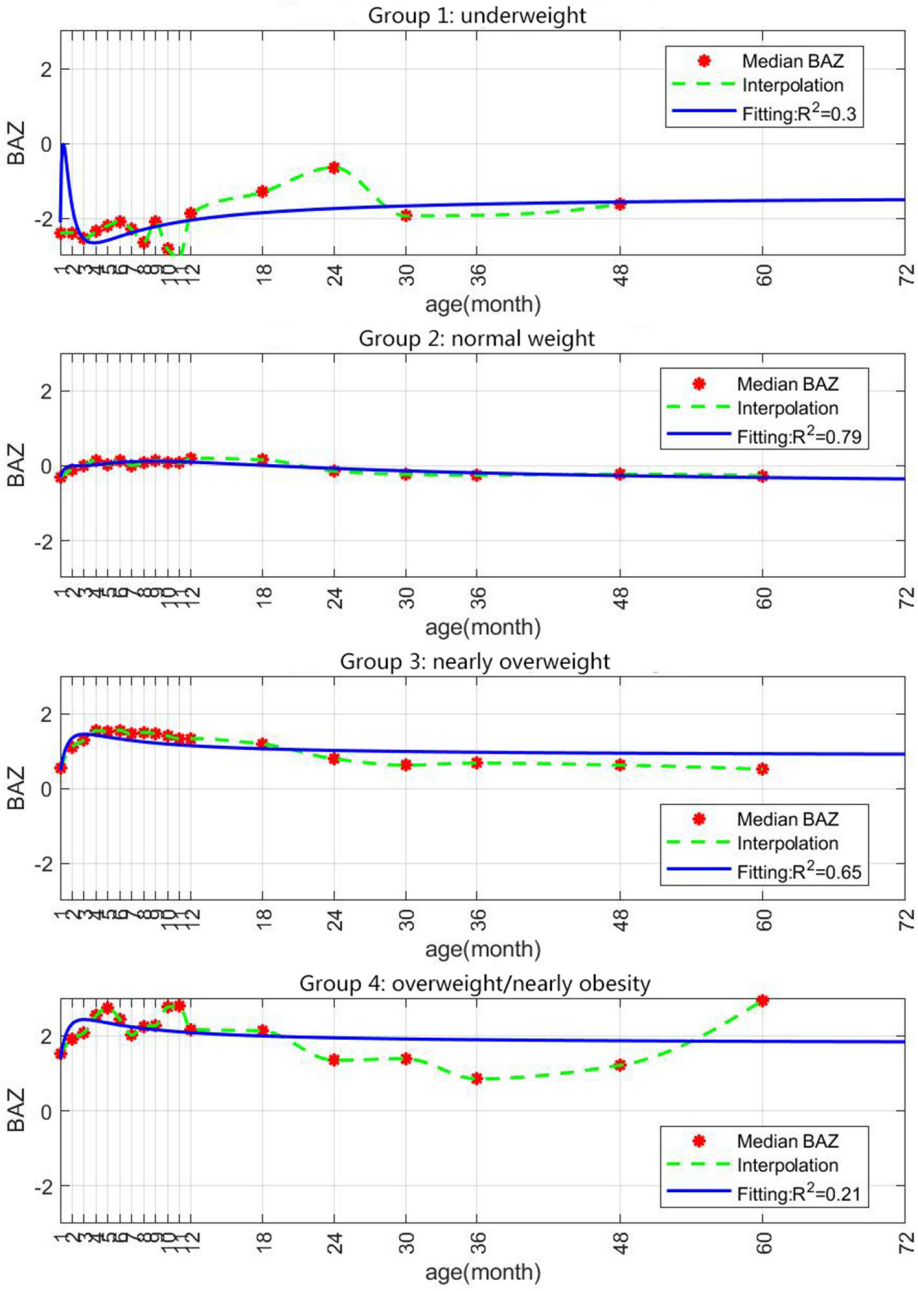
Prediction of growth in BAZ in the different study groups based on data of 23,871 children. SGA infants were assigned at 0–12 months to the following groups by BAZ (BMI-for-age Z-scores): underweight (BAZ < −2), normal weight (−1 to 1), nearly overweight (1 to 2) and overweight/nearly obese (BAZ > 2).

The incidence of obesity at school age (6–7 years) was 12.1% according to the BMI distribution (BAZ). The fitted curve shows the distribution of BAZ in different groups of children from infancy to school age. As seen in Figure 3, SGA infants showed a catch-up trend till 12–18 months of age, but their BAZ remained stable after that. Former SGA babies who were underweight or normal weight at 6–7 years of age had remained so throughout the period from birth to 6–7 years. Even though SGA infants who developed obesity had a stage of weight loss in their early life, they had obesity by the age of 2 years. From the point of view of the fitting coefficient, the R2 values of group 1 and group 4 in Figure 3 are lower, which may be affected by extreme values. If there are too many extreme values in the measurement indicators, R2 will be low. It is not difficult to see from the data results that the two groups of Group 1 and Group 4 are indeed more affected by the extreme values.

### Accelerated Increase in BMI in Early Childhood and the Occurrence of Obesity in School-Age Children

For the second part of the analysis 6,959 children were included of which 36.3% were male. Baseline difference between included and excluded children is presented in Table 1.

**Figure 4:**
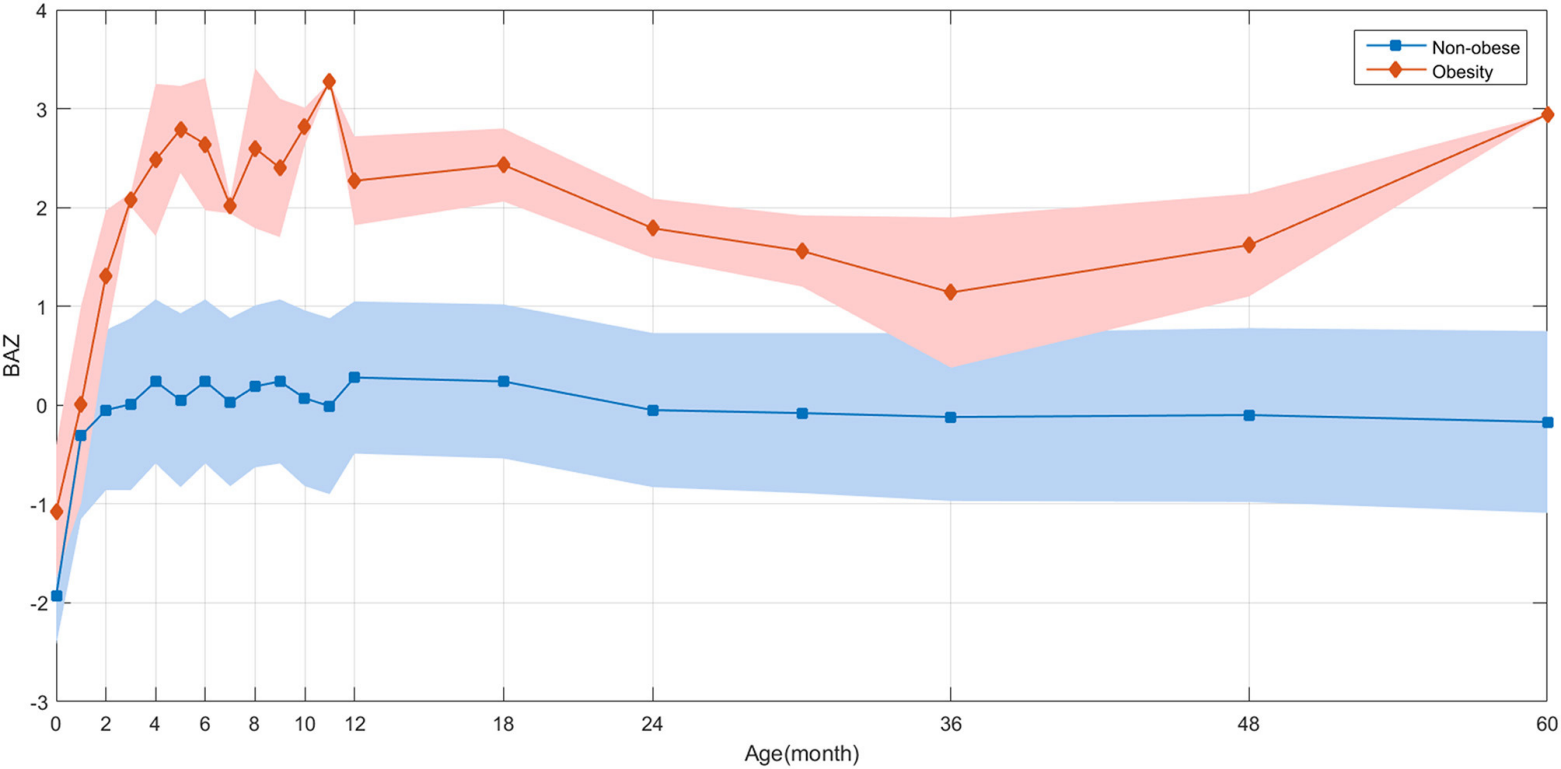
BAZ growth trajectories during childhood based on data of 6,959 children. A retrospective observation of the distribution of BAZ for children with and without obesity is demonstrated.

**Figure 5:**
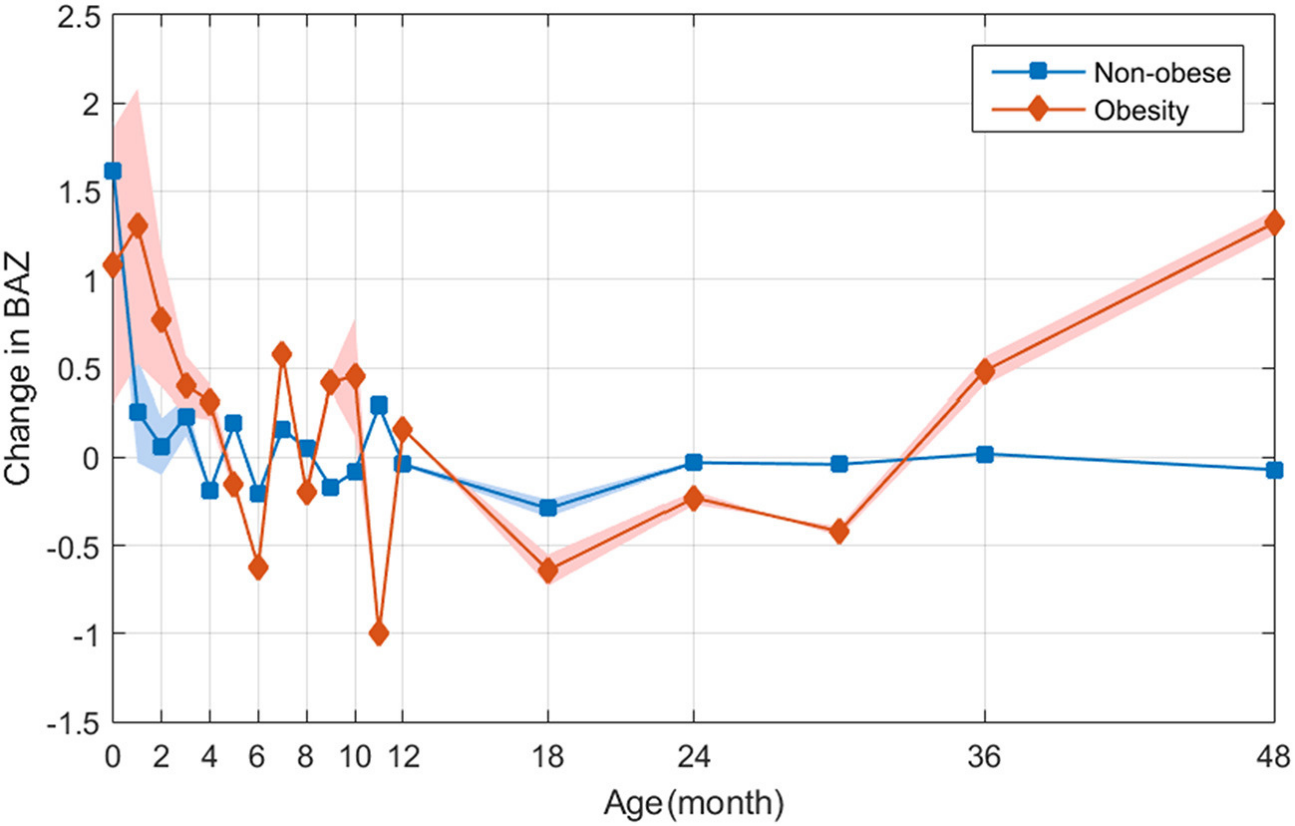
Retrospectively observation of the distribution of ΔBAZ between children with and without obesity based on data of 6,959 children.

The average annual BMI and BMI changes remained stable in formerly underweight or normal weight infants. However, children with overweight or obesity had exhibited a sustained increase in BMI in early childhood. The annual changes in BMI in the normal-weight group were more stable compared to infants who had obesity. Also, in infants who had obesity there was extremely rapid catch-up growth in the initial months with BAZ reaching >1 at 1 month of age (Figure 4). For school-aged children who had obesity who had been SGA newborns, the most significant increase in BMI occurred before 6 months of age (Figure 5). The annual change in BAZ remained positive (although at a low rate) and unstable thereafter, leading to a high rate of obesity.

We divided the former SGA infants into two groups based on the presence of obesity and analyzed their BAZ trajectories. The average BAZ change in the “non-obesity” group (i.e., infants who were underweight/normal weight) remained stable. However, the children with overweight or obesity continued to exhibit an increase in BMI from early childhood (Figure 5).

By analyzing the early BMI changes, we found that the fastest growth rate of SGA infants occurred before 6 months of age, and that an accelerated rate of BMI increase persisted throughout childhood.

## Discussion

Several studies have shown that rapid weight gain in the early postnatal period is related to the occurrence of obesity later in life (17–20). Our study with a large sample of 23,871 full-term SGA newborns suggests that they experience catch-up growth after birth but at different rates. Most SGA infants had below-normal heights, but their weights were more prone to individual variations as compared to normal children. In general, former SGA newborns develop overweight and obesity while growing in height at a slower pace as compared to normal infants.

Our study demonstrates that SGA infants developed overweight and obesity in early childhood i.e., before 6 months of age. Also, children who had obesity at the age of 1 year continued to have obesity at school-age. After the initial stage of rapid weight gain, BMI showed strong instability and even negative growth after 6 months of age. However, after 36 months of age, BMI continued to increase at a low but positive rate, resulting in obesity in this group of children. However, in the children with normal weight at school-age, the BMI remained stable throughout childhood. We also found that the prediction trend of the fitted curve of former SGA infants who were overweight/nearly obese differs from the development curve of nearly overweight infants. Unlike infants who developed obesity, the weight of nearly overweight infants returns to normal with age.

The incidence of obesity in our study was 12.11%, a rate similar to the Chinese school-age children's overweight and obesity rate (19.41%), but lower than that reported in a study on school-age children (21, 22). This discrepancy can be explained by the special attributes of the groups. SGA infants with failed catch-up growth continue to have prolonged low developmental levels (23). Rapid BMI increases have been shown to occur in early childhood (24, 25), and this is consistent with the age at which we observed the fastest weight gains in our cohort.

According to the Developmental Origins of Health and Disease (DOHaD) theory, the risk of obesity is higher in individuals with lower birth weights or who are SGA (26, 27). Our study including the entire age range from birth to school age showed that the presence of early obesity considerably increases an individual's risk of overweight and obesity in the future. Also, dynamic BMI changes may be important to identify the future risk of obesity. In our analysis, there was a sharp rise in BAZ at 48–60 months. Studies indicate that BMI in children decreases at 1 year of age which shifts to an increase before adulthood. This concept of “BMI rebound,” its timing and subsequent risk of obesity has been studied in literature. Evidence from studies on “BMI rebound” suggests that children with earlier or significant BMI rebound have a much higher risk of developing childhood obesity (28, 29). Furthermore, the magnitude of the effect can be substantial (>3 body mass index units at 18–21 years) for those undergoing early (<5 years of age) compared with late (>7 years of age) rebound (29, 30).

Our study on SGA infants concurs with the results of studies conducted in the general pediatric population (9–12, 25). Previous research has shown that children who develop obesity early in life have less than a 20% chance of regaining their normal weight during adolescence and that this rate decreases further with age (9, 25). The dynamic BMI increases do not end after puberty, and they may continue into adulthood. One limitation of our study was that we followed up the participants only till school-age, and could not conduct a more comprehensive assessment of the risk of obesity during puberty or adulthood. Also, in the second part of the analysis, the gender ratio was not 50% with underrepresentation of male children in our analysis. This, however, could have been due to sampling error and was beyond our control. Also, studies indicate that in the Chinese population girls are at a higher risk of being born SGA as compared to boys (31). Another limitation of our study is our inability to rule out the influence of dietary habits and energy intake on weight gain. In the initial study design, the research team believed that children before 12 months of age are mainly fed with breast milk, milk powder and simple complementary foods. Compared with the diet structure after 1 year of age, the initial diet is relatively simple. Therefore, more detailed data on the frequency of feeding and energy intake of SGA children were not retained at the time of data collection. Our data also demonstrated instability in the rate of weight gain at 6–10 months of age. The unstable BAZ during this time period can be due to two possible reasons. First, the age corresponding to the negative value of BAZ in the data chart is the period when the infant starts to climb, stand, and walk with support, which is the period of increased energy consumption. Secondly, the time corresponding to this month's age is also a critical period for starting to add supplementary food which brings in more uncontrollable factors. Lastly, while we used the WHO criteria for classification of SGA babies and defining postnatal obesity, since our study was conducted specifically on only Chinese population the classification may not be fully appropriate for the study population. Thus, the results should be interpreted with caution.

In summary, our results show that the amount of early weight gain after birth in full-term SGA infants can influence not only their physical development but also their risk of developing long-term metabolic diseases such as obesity. SGA infants should be closely monitored from an early stage, and childhood obesity screenings should be expanded to include infants. The risk of early obesity is high during the catch-up growth period, and once an infant develops obesity, the condition will probably persist and have a long-term impact. The most significant weight gain in SGA term infants occurred during their first year after birth. Furthermore, the BAZ scores were not persistently high showing signs of decrease followed by a rebound at 48–60 months. Obesity that occurs early and is accompanied by a sustained increase in BMI may lead to severe obesity.

## Data Availability Statement

The raw data supporting the conclusions of this article will be made available by the authors, without undue reservation.

## Ethics Statement

The studies involving human participants were reviewed and approved by Ethics Committee of Shanghai Children's Hospital Affiliated to Shanghai Jiaotong University School of Medicine. Written informed consent to participate in this study was provided by the participants' legal guardian/next of kin.

## Author Contributions

DW was a major contributor during the writing of the manuscript. JZ and DW analyzed the data. XW, YH, ML, FS, HS, HLa, CG, HLi, TinL, JX, LJ, XH, TiaL, GYa, and GZ collected the data. JC and GYu supervised the study. All authors read and approved the final manuscript.

## Conflict of Interest

The authors declare that the research was conducted in the absence of any commercial or financial relationships that could be construed as a potential conflict of interest.
